# Distinguishing the Visual Working Memory Training and Practice Effects by the Effective Connectivity During n-back Tasks: A DCM of ERP Study

**DOI:** 10.3389/fnbeh.2019.00084

**Published:** 2019-04-17

**Authors:** Chun-Chuan Chen, Ju-Che Kuo, Wei-Jen Wang

**Affiliations:** ^1^Department of Biomedical Sciences and Engineering, National Central University, Taoyuan, Taiwan; ^2^Institute of Cognitive Neuroscience, National Central University, Taoyuan, Taiwan; ^3^Department of Computer Science and Information Engineering, National Central University, Taoyuan, Taiwan

**Keywords:** visual working memory training, neuroplasticity, executive control network, dorsal attention network, effective connectivity

## Abstract

Visual working memory (WM) training and practice can result in improved task performance and increased P300 amplitude; however, only training can yield N160 enhancements. N160 amplitudes are related to the spatial attention, the detection of novelty and the inhibitory control, while P300 amplitudes are related to the selective attention. Therefore, it could be speculated that the mechanisms underlying N160 and P300 production may differ to accommodate to their functions. Based on the different N160 engagements and different functional roles of N160 and P300, we hypothesized that the effects of visual WM training and practice can be dissociated by their brain effective connectivity patterns. We compared different neural connectivity configurations for the main task-related brain activities including N160 and P300 during the visual three-back task in subjects after visual WM training (the WM group) and after repetitive task practice (the control group). The behavioral result shows significantly greater improvement in accuracy after training and suggests that visual WM training can boost the learning process of this simple task. The N160 peak amplitude increased significantly after training over the anterior and posterior brain areas but decreased after practice over the posterior areas, indicating different mechanisms for mediating the training and practice effects. In support of our hypothesis, we observed that visual WM training alters the frontal-parietal connections, which comprise the executive control network (ECN) and the dorsal attention network (DAN), whereas practice modulates the parietal-frontal connections underpinning P300 production for selective attention. It should be noted that the analytic results in this study are conditional on the plausible models being tested and the experimental settings. Studies that employ different tasks, devices and plausible models may lead to different results. Nevertheless, our findings provide a reference for distinguishing the visual WM training and practice effects by the underlying neuroplasticity.

## Introduction

Working memory (WM) is a system that is required to maintain and manipulate information to perform complex reasoning or learning tasks (Baddeley, 2003). It is believed that WM capacity relates to learning ability (Willis and Schaie, 2009) and can be enhanced through WM training. Behavioral measures indicate that WM training improves WM functions and task performance (Buschkuehl et al., 2008; Jaeggi et al., 2008; Harrison et al., 2013; Redick and Lindsey, 2013; Thompson et al., 2013; Spencer-Smith and Klingberg, 2015). Furthermore, neurophysiological studies provide convincing evidence of neuronal effects following WM training (for reviews, see Buschkuehl et al., 2012; Constantinidis and Klingberg, 2016). The n-back task is the most used task to investigate the neuronal correlates of WM functions and training effects because higher-order control processes, including maintenance, rehearsal and manipulative processes like updating of memory contents, can be accessed through this task (Cohen et al., 1997; Owen et al., 2005; Schneiders et al., 2011). When measured with the event-related potentials (ERPs) during visual n-back task, N160, a negative peak evoked at around 160 ms after stimulus, and P300, a positive peak at around 300 ms after stimulus, were consistently enhanced after WM training over the frontal-central and the temporal-parietal area (Zhao et al., 2013; Covey et al., 2018). When measured with neuroimaging techniques for the local activities after WM training, it has been reported that the middle frontal gyrus (MFG), superior frontal gyrus (SFG) and intraparietal sulcus (IPS) were involved during visual n-back tasks (Olesen et al., 2004; Klingberg, 2010; Schneiders et al., 2011). Recently, Thompson et al. (2016) further tested the functional anatomy after intensive WM training using fMRI. They reported that WM training enhanced the functional connectivity within both the executive control network (ECN)—which comprises the dorsolateral frontal gyrus, dorsomedial frontal gyrus, and IPS—and the dorsal attention network (DAN)—which comprises the human frontal eye fields, SFG, and superior parietal lobe. In summary, WM training can result in improved task performance and enhanced N160 and P300 amplitudes during visual n-back tasks (McEvoy et al., 1998; Zhao et al., 2013). Moreover, the WM training effects are possibly mediated by the frontal-parietal and parietal-occipital network alternations (Kundu et al., 2013; Thompson et al., 2016). However, the analysis of the characteristics of the ERPs’ components of N160 and P300 has not been used to specifically address the WM training effects on the frontal-parietal connections. Previous studies tested only the oscillatory activities measured with EEG and their coupling in the frontal-parietal network during WM tasks (Sauseng et al., 2005; Astle et al., 2015; Ewerdwalbesloh et al., 2016). It remains unclear whether N160 and P300 changes after WM training are associated with the frontal-parietal network alternations.

WM practice can enhance the WM function too. The effects of WM practice are similar to that of WM training in two ways: an improved task performance (Adam and Vogel, 2018) and an enlarged P300 amplitude (McEvoy et al., 1998; Zhao et al., 2013). However, no studies have ever reported an increase in N160 magnitude after practice. In fact, in Ahonen et al. (2016) study, they demonstrated that the latency and amplitude of M160, a magnetoencephalographic equivalent to N160 when measured with magnetoencephalography, remain stable at the group level across four consecutive measurements. Indeed, the functional roles of N160 and P300 may not be identical, although both are related to attention. Previous studies have suggested that the enhanced N160 amplitudes are related to the selective allocation of spatial attention (McEvoy et al., 2001), the detection of novelty or mismatch and the cognitive control (Folstein and Van Petten, 2008), while the enhanced P300 amplitudes are related to the selective attention (Linden, 2005; Duncan et al., 2009). Therefore, it could be speculated that the underlying mechanisms for N160 and P300 generation may differ to a certain degree to accommodate to their functions. Regarding the neuronal correlates of P300, no studies thus far have investigated the network modifications concerning P300 after WM practice using n-back tasks. The mechanism of P300 generation has been studied mainly using the oddball paradigm and conclusions have been relatively inconsistent (Crottaz-Herbette and Menon, 2006; Chen et al., 2014). For instance, it has been reported that the parietal-frontal neural network of the anterior cingulate cortex (ACC), dorsolateral prefrontal cortex (DLPFC) and inferior parietal lobule (IPL) constitutes a common mechanism for P300 production in the oddball task (Huang et al., 2005; Chen et al., 2014). Crottaz-Herbette and Menon (2006) identified the left premotor area (PMA) and primary sensory areas, in addition to ACC and IPL, to form a network underlying the generation of P300 (Crottaz-Herbette and Menon, 2006). In brief, P300 can be modulated after WM practice and training and is generated by a distributed network.

Therefore, based on the difference in N160 engagements between WM training and practice and different functional roles of N160 and P300, we hypothesized that the effects of VM training and practice can be dissociated by their brain effective connectivity patterns associating with the main task-related brain activities including N160 and P300 components during the visual three-back task. Specifically, we tested whether the visual WM training could lead to neuroplasticity of ENC and DAN while practice is mainly about altering the effective connections underpinning P300 production for selective attention. Finally, we studied the visual WM training-specific modulations on the effective connectivity that may be responsible for the fluctuations in cortical signals after training.

## Materials and Methods

### Participants and Training

**Figure 1:**
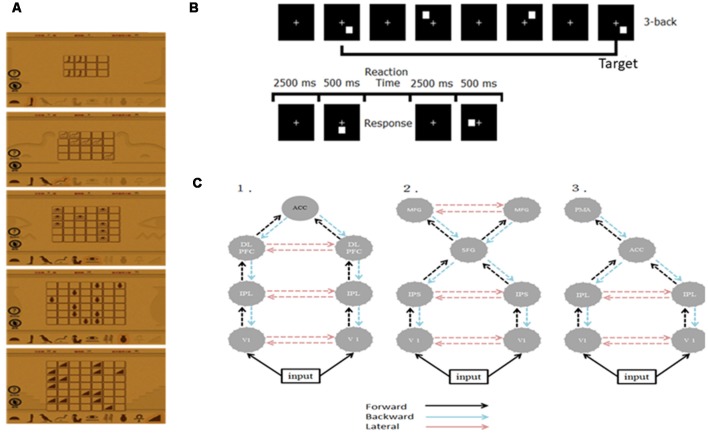
**(A)** Illustration of the training material. **(B)** The experimental paradigm of the 3-back task. **(C)** Architectures of the three plausible DCMs. ACC, anterior cingulate cortex; DLPFC, dorsolateral pre-frontal cortex; IPL/S, intraparietal lobe/sulcus; V1, primary visual cortex; MFG, middle frontal gyrus; SFG, superior frontal gyrus; PMA, premotor area.

We recruited 20 right-handed, healthy graduate students and randomly divided them into two groups—the WM (*n* = 10; mean age = 23.9; two females) and control groups (*n* = 10; mean age = 24.5; one female). We instructed the WM group to log in a public website and play a memory matching online game, Memory Matrix 3, as their WM training task^1^ at home. This game comprises two phases, namely the memory and the retrieval phases. We instructed the participants to memorize the shapes and positions of X pairs of objects that were arranged in a matrix within 30 s, after which we covered these objects. In the retrieval phase, we instructed the participants to randomly select an object in the matrix and find its match according to their memory. The difficulty level of the task is proportional to the value of X and the initial level of task difficulty in each training section is subjective to the participant’s performance. Figure 1A shows the training materials. The training strength was 30 min per day, 5 days a week for 3 weeks. On a daily basis, we check the logbook of each participant in the WM group to see if they comply with the prescribed training dose. A reminder was given to those who have missed one training (i.e., 30 min) such that all subjects adhered to the training plan. By the end of the training, all WM subjects reached the most difficult level (*X* = 7). We did not conduct any training for the control group. The study was approved by the Research Ethics Committee of National Taiwan University (approval number: 201311ES020) in accordance with the ethical principles of the Declaration of Helsinki and all subjects have signed an informed consent form.

### Visual Three-Back Task

We employed the visual 3-back task to probe the neuronal correlates of visual WM. During the visual 3-back task, we presented a series of stimuli consecutively and participants had to decide whether the presented stimulus matched the stimulus that was presented three positions back in the sequence. A total of 80 targets and 160 nontargets were presented randomly in sequence every 3 s (stimulus length = 500 ms; interstimulus interval = 2,500 ms). A response was required for every stimulus and participants responded manually by using their right hand to press the space key of a standard computer keyboard. Figure 1B illustrates the paradigm.

### Electroencephalography Acquisition and ERP Analysis of N160 and P300

All subjects completed the 3-back task four times: before training/practice (W0), after a week (W1), 2 weeks (W2) and 3 weeks (W3) from recruitment. When the participants were performing the spatial three-back task, we acquired a 16-channel EEG (F3, Fz, F4, C3, Cz, C4, T7, T8, P7, P3, Pz, P4, P8, Oz, O1, O2) with 2,000 Hz sampling rate. To obtain the most significant WM training effects, we analyzed only the EEG data acquired at W0 and W3 in this study. We processed the EEG data using SPM12 and a standard EEG pre-processing procedure to obtain the ERPs. We band-pass filtered the EEG data (0.01–58 Hz) and epoched the trials with a peristimulus window of −2,000 to +2,500 ms, where the time zero indicated the presence of the stimulus. We removed electrooculography contamination trials when EEG amplitude was greater than 100 mV from the epoched data, and those electrooculography-free trials were divided into correct and incorrect trials depending on the participant’s response. Only the correct trials were further filtered with the 30-Hz low-pass filter, down-sampled to 250 Hz, baseline-corrected (−200 to 0 ms), and averaged across trials (for a detailed description of this processing procedure see Kundu et al., 2013). The resulting mean ERPs in both the WM and the control groups were first examined phenomenally to identify the N160 (negative peak around 150–220 ms) and P3 (positive peak around 250–500 ms). In addition, mean P300 signal changes were obtained by averaging over a period of post-stimulus time from 250 to 600 ms, because the peak value of P300 exhibited large individual differences (Zhao et al., 2013). Finally, the ERPs were analyzed with the effective connectivity of dynamic causal modeling (DCM) of ERPs (David and Friston, 2003; David et al., 2006).

### The Analysis of Effective Connectivity

We analyzed the effective connectivity during visual 3-back task using an established method, DCM for ERPs (David and Friston, 2003; David et al., 2006). The theoretical background can be found in David et al. (2006) and David and Friston (2003). Here, we briefly introduce the concepts of DCM for ERPs. DCM for ERPs aims to explain ERPs using a network of coupled cortical sources based on a biologically plausible model, the neural mass model. In DCM, the neural populations receive exogenous perturbations that alter their coupling strength and lead to the changes of ERPs. Estimates of the coupling strength alternation in the presumed network given the data and the experimental manipulation allow the understanding of where and how much the neural network has been changed. Henceforth, DCM for ERPs is a hypothesis-driven method and the first step for applying it is to form a hypothesis and construct the plausible models.

In this study, we used only neuronal responses recorded between −300 and 1,000 ms because these signals can capture the cortical responses for N160 and P300 while excluding the movement-related responses and applied the two-step strategy to reduce the number of potential model combinations (Chen et al., 2014). First, we specified three plausible models to test whether WM training and practice effects were mediated by various mechanisms based on the previous studies discussed in the “Introduction” section (Huang et al., 2005; Crottaz-Herbette and Menon, 2006; Schneiders et al., 2011; Chen et al., 2014; Thompson et al., 2016). The three models shared the bilateral primary visual cortex (V1), but they differed in some higher areas and connections. Model 1 was a distributed parietal-frontal network comprising bilateral DLPFC [−34 25 29; 37 23 30], IPL [−37 50 46; 46 46 41], and ACC [1 4 29] for P300 generation during the oddball task (Huang et al., 2005; Chen et al., 2014). Model 2 engaged ECN and DAN seen after intensive WM training (Schneiders et al., 2011; Thompson et al., 2016). Model 3 included bilateral IPL [−40 −38 56; 46 −26 32] and left PMA [−32 −16 −64] derived from the result of Crottaz-Herbette and Menon (2006) for attentional control. The source locations in these models (in Talairach coordinates) were taken from the cited studies that motivated the models as the initial guesses. The modulation resulting from WM training and practice in these networks was assumed to be reciprocal in all connections at this step. Figure 1C shows the connection architectures of three plausible models. Second, after establishing the most likely connection models for the WM and control groups, we further examined where the specific modulation at W3 occurred. We altered the modulation as forward (F), backward (B), or lateral (L), thereby constructing five additional models (denoted as F, B, FB, FL, and BL) that contrasted with the modulations in all the connection models that resulted from the first step (i.e., FBL). Bayesian inversion of DCM provided the estimates of these model parameters and the optimal source locations for each individual. After Bayesian inversion of DCM, Bayesian model selection was employed to identify the optimal models among those being tested at the individual and the group levels (Stephan et al., 2009, 2010). Details on this standard procedure of the DCM analysis can be found in several previous studies (Garrido et al., 2007, 2008; Chen et al., 2014).

### Statistical Tests

The behavioral data of task performance, including accuracy and reaction time, were entered into a 2 × 4 repeated measures ANOVA with two factors, namely group (WM and control) and session (W0 to W3), to test whether WM training affected accuracy and reaction time and whether these effects relied on the duration of training. To test whether the WM training altered neuronal activities, the ERPs of N160 and P300 were entered into a 2 × 2 × 16 repeated measures ANOVA with three factors, namely group, session, and electrode. Finally, the *post hoc* test was performed to assess any differences among the factors. The significance level was set at *p* < 0.05 after correction for multiple comparisons (Bonferroni–Dunn correction). For the DCM analysis, after identifying the optimal model by using Bayesian model selection, we tested the modulatory effect of the experimental manipulation by performing a *t*-test to identify significant modulatory connection parameters.

## Results

### Behavioral Data and ERPs

**Figure 2:**
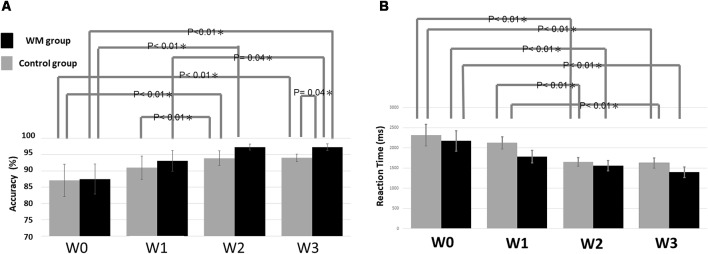
Statistical results of **(A)** the accuracy and **(B)** the reaction time. The error bars indicate the standard error of the mean (SEM).

Figure 2A summarizes the statistical results of the accuracy. The ANOVA result indicated a significant factor of session (*F* = 19.33, *p* < 0.05) and a significant interaction between session and group (*F* = 6.04, *p* < 0.05). The *post hoc* test revealed that the WM group displayed significantly higher accuracy at W3 than did the control group (*p* = 0.04). The nonsignificant group effect at W0 confirmed that the two groups did not differ significantly at baseline. Regarding the impact of session on accuracy, the WM group shows a significant improvement in accuracy between W0–W2 (*p* < 0.01), W1–W3(*p* = 0.04) and W0–W3 (*p* < 0.01) whereas the control group exhibits a significant improvement in accuracy between W0–W2 (*p* < 0.01), W1–W2 (*p* = 0.01) and W0–W3 (*p* < 0.01).

With respect to the reaction time, there is only a significant factor of session (*F* = 16.011, *p* < 0.05). Figure 2B showed that the reaction time was significantly shorter between W0–W2 (*p* < 0.01) and W0–W3 (*p* < 0.01) for both groups, and additional W1–W2 (*p* < 0.01) and W1–W3 (*p* < 0.01) significance for the control.

Session was a significant factor for the N160 amplitudes (*F* = 2.920, *p* < 0.05), and there were significant interactions between group, session, and electrode (*F* = 4.06, *p* < 0.05). When compared with the control group, the WM group displayed a significant increase in N160 amplitude at P8 (*p* = 0.046), T8 (*p* = 0.006), and C4 (*p* = 0.038) at W3 after training. Specifically, the N160 peaks over P8 revealed different patterns for training and practice. At W3, the N160 amplitude of the WM group increased (*p* = 0.008) but that of the control group decreased (*p* = 0.017) when compared with those at W0.

**Figure 3:**
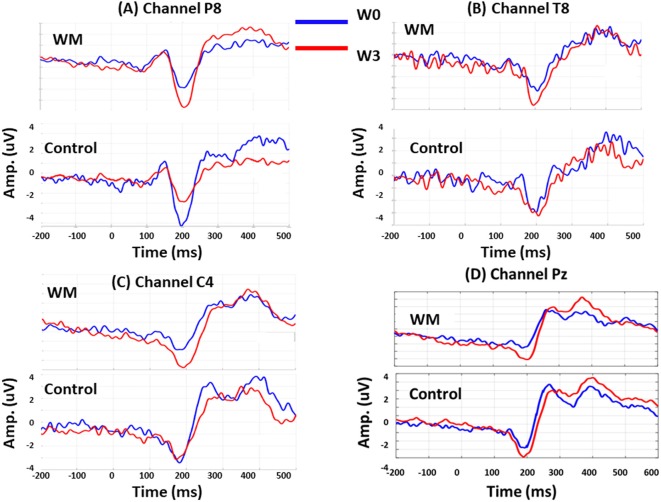
Group-specific mean time courses of the event-related potentials (ERPs) at W0 (red lines) and W3 (blue lines) over channel P8 **(A)**, T8 **(B)**, C4 **(C)** and Pz **(D)**.

After WM training, both the WM and control groups exhibited a greater P300 peak amplitude over Pz at W3 compared with that at W0, but these results were nonsignificant. After averaging the P300 amplitude from 250 to 600 ms, we obtained a significant result from the interaction between electrode and session (*F* = 3.14, *p* < 0.05). Both groups increased the mean P300 amplitude over Pz at W3 (*p* = 0.045 and *p* = 0.037 for the WM and control groups, respectively), but this enhancement did not differ between the two groups. Figure 3 displays the group-specific mean time courses of the ERPs (averaged across participants) over channels P8 (A), T8 (B), C4 (C), and Pz (D) at W0 and W3.

### Inferences on Model Space

**Figure 4:**
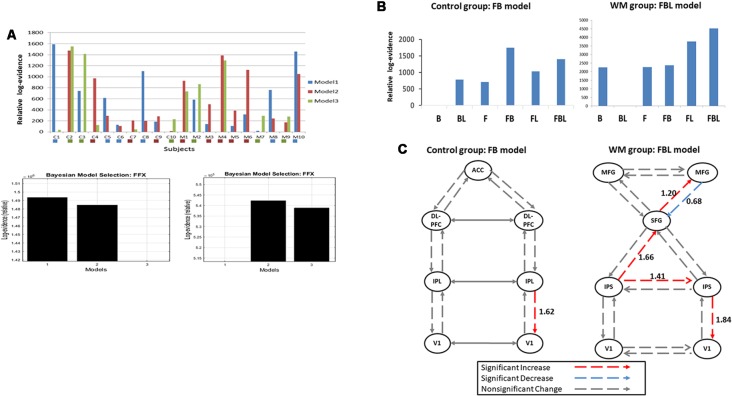
**(A)** Model selection results at the individual level (upper panel) and the group level for the control (left, lower panel) and the working memory (WM) training (right, lower panel) groups. C1–10: the control group; M1–10: the WM group. The colored box under each subject indicates the subject-specific winning model. **(B)** Model selection results of the modulatory effects for the control (left) and the WM training groups (right). B, backward; F, forward; L, lateral. **(C)** Group-specific modulations at W3. Left, the control group; Right, the WM group.

We inverted three plausible DCM models for each participant (Figure 1C). Figure 4A indicates the Bayesian model selection results at the individual level (upper panel) and the group level (lower panel). In the WM group, three, five, and two participants had Model 1, Model 2, and Model 3 as their optimal model, respectively. These models were optimal for four, three, and three participants, respectively, in the control group. At the group level, the Bayesian model selection results indicated that Model 2 for the WM group (Figure 4A, right lower panel) and Model 1 for the control group (Figure 4A, left lower panel) were the optimal models. Having identified the best model, we further investigated the modulation mechanism by comparing the optimal model against five derivative models (see “The Analysis of Effective Connectivity” Section). For the WM group, the FBL model under the ECN and DAN architecture can best explain the training-induced alternations in the effective connectivity (Figure 4B; right). For the control group, the practice effects were medicated by the FB loop in the parietal-frontal effective connection (Figure 4B; left).

### Inference on Modulatory Effects

The *t*-test was applied to the modulation parameter matrices of the optimal models for participants in the same group to assess modulation effects at W3. Figure 4C illustrates the statistical results of the modulatory parameters. In the WM group (Figure 4C; right), five modulations were statistically significant after training, including the forward modulations from left IPS to SFG and from SFG to right MFG, the lateral modulation between the two IPSs, and the backward modulations from right MFG to SFG and from right IPS to V1. All but one of the five significant modulations were excitatory. The exception was the backward modulation from right MFG to SFG (blue line, Figure 4C), which was significantly down-regulated after visual WM training. By contrast, the control group had only one significant excitatory feedback modulation from right IPL to right V1 at W3 in the parietal-frontal network underlying P300 production (Figure 4C; left).

## Discussion

In this study, we examined the differences in the behavioral data and the neuronal signals during visual three-back task after visual WM training and repetitive task practice. We found that: (1) behaviorally, both the WM and control groups improved their task performance and the accuracy improvement of the WM group after training was significantly greater than that of the control group; (2) both visual WM training and practice enhanced the P300 amplitudes, but only training can yield an increased N160 peak amplitude; and (3) the underlying neural connectivity patterns for visual WM training and practice are not identical. Visual WM training alters the frontal-parietal connections for the executive control and attention while WM practice engages the parietal-frontal connections with the ACC for selective attention.

### Visual WM Training Boosts the Learning Process of Simple Tasks

Accuracy and reaction time improved with the retest times in both groups, indicating that practice can enhance task performance. The result that the accuracy improvement of the WM group at W3 was significantly greater than that of the control group indicates a further beneficial effect on task performance after training. Interestingly, we observed that from W2 to W3, the mean accuracy of both groups did not have a significant increase (Figure 2A). The reason that the accuracy at W3 was significantly greater in the WM group than in the control group is because of the decrease in the between-participant variability [i.e., a smaller standard error of the mean (SEM)] in the control group. In other words, practice increased the stability of task performance in the control group at W3, likely through reducing the chance of success by guessing. By contrast, the WM group reached a stable performance (a small SEM) with a mean accuracy greater than 90% at W2. As a stable and accurate performance is an indication of the learning endpoint, our result implies that visual WM training can boost the learning process of this task.

### Training Effects on the N160 Peak Amplitude

N160 peak amplitude was reported to be related to the selective allocation of attention during a visual-spatial n-back task (McEvoy et al., 2001) and WM training can augment the N160 amplitude over the frontal-central area (Covey et al., 2018) and the right temporal-parietal area (Zhao et al., 2013). In a review article, it was proposed that the N160 elicited by visual stimuli can be divided into functionally distinct subcomponents: the frontal-central (anterior) components related to the detection of novelty or mismatch and the cognitive control (encompassing response inhibition, response conflict, and error monitoring) and the posterior N160 related to some aspects of visual attention (Folstein and Van Petten, 2008). A greater N160 amplitude is related to more neuron-related cognitive control (Xiao et al., 2019), in particular, the inhibitory control for irrelevant information and a diminished N2 amplitude was seen in the error trials, suggesting that the impaired cognitive control leads to errors (Kok et al., 2004; Falkenstein, 2006; O’Connell et al., 2009). In this study, we showed that, only after visual WM training but not practice, the N160 peak amplitude increased significantly at W3 over C4 (frontal-central area), T8 and P8 (temporal-parietal area). In contrast, in the control group, we observed a decreased N160 amplitude over P8 at W3 and a stable N160 amplitude over the other channels. In summary, visual WM training additionally modulates the anterior N160 components related to the detection of novelty and the cognitive control whereas practice reduces the posterior N160 amplitude to decrease visual attention. Hence, N160 difference between the WM and control groups may indicate different mechanisms for mediating the training and practice effects.

### Visual WM Training and Practice Separately Modulate the Neuronal Connections

The Bayesian model selection result revealed that visual WM training and practice can lead to different levels of neuroplasticity; visual WM training alters the frontal-parietal connections, comprising the ECN and DAN, with bilateral MFG on top of the neural hierarchy, whereas WM practice engages the parietal-frontal connections with ACC on top. The ECN and DAN were thought to underlie WM and WM training can increase their functional connectivity (Thompson et al., 2016). In this study, we further demonstrated that ECN exerted a modulatory suppression on the DAN through the feedback loop from right MFG to SFG after visual WM training. This modulatory suppression may reflect the mechanism used by MFG as a neuronal gatekeeper to prevent irrelevant information from entering the WM system (McNab and Klingberg, 2008). Moreover, we observed significant excitatory modulation effects from SFG to right MFG and from right IPS to right V1 after WM training. Schmicker et al. (2016) reported that there was a lateralized enhancement of the activity over the right MFG and the occipital visual area during WM tasks measured using fMRI after attentional filtering training. The authors interpreted the increase in activity as a strengthened neuronal loop for the effective control of visual information processing (Schmicker et al., 2016). Our results of the excitatory inferences from SFG to right MFG and from right IPS to right V1 after WM training may disclose the possible production mechanisms for this strengthened neuronal loop.

Regarding the WM practice effect, it was thought that WM practice can lead to a greater ability to filter irrelevant information (Berry et al., 2009) and increased neural efficiency, particularly in task-relevant sensory areas, such as visual areas (Garavan et al., 2000). In this study, we found a significant excitatory inference from the right IPL to right V1 in the parietal-frontal connections for P300 generation after WM practice. Given that the stimuli and task were not changed, the result of the excitatory IPL-V1 connection may indicate a better neural efficiency after WM practice such that the identical stimulus leads to a greater response.

### Both WM Training and Practice Enhance the P300 Amplitude

The P300 was reported to be significantly enhanced after WM updating function training (Zhao et al., 2013) and higher-order cognitive strategy training (Motes et al., 2014). Both studies obtained the mean P300 signal changes by averaging over a period of poststimulus time from 250 to 600 ms (Zhao et al., 2013; Motes et al., 2014) because the peak value of P300 exhibited large individual differences (Zhao et al., 2013). In our study, the enhancement of P300 peak amplitude after training was nonsignificant in both the WM (*p* = 0.051) and control groups (*p* = 0.053) due to substantial between-subject variabilities. We obtained a significant P300 increase at Pz in both groups only after averaging over time. This result, together with the previous reports, suggest that the analysis of WM training effects should take into account a longer period of brain dynamics to best capture the underlying brain mechanism. As the functional role of P300 is related to the attention, our result of P300 enhancement in both groups demonstrates that attention modulation can be yielded through both practice and training, although the underlying mechanisms are not identical.

### Nonsignificant P2 Modulation After WM Training and Practice in Visual 3-Back Task

P2, the positive peak around 200 ms, is part of memory information process related to memory updating (Lenartowicz et al., 2010; Yuan et al., 2016) and is a potential candidate for investigating the WM training effects. P2 amplitude decreased in the error trials (Xiao et al., 2019) and after WM training in 2-back task (Zhao et al., 2013) but increased in the frontal-central sites in the 1-back and 2-back conditions after exposure to long-term stress (Yuan et al., 2016) or high altitude (Ma et al., 2019). In this study, neither increase nor decrease in P2 peak can be observed in both groups. At first glance, our result of nonsignificant P2 modulation after training seems to contradict the previous reports. However, none of the previous studies employed the 3-back task. As memory load is an important aspect of evaluating WM function, our result only indicates that the 3-back task is not suitable for probing the P2 modulation after WM training/practice. Whether P2 is modulated after WM training or practice remains inconclusive in this study.

### Considerations on the Sources of EEG Signals and the DCM Results

In this study, we measure the scalp EEG but examine the connectivity alternations among cortical areas after WM training and practice. Given that the spatial resolution is limited in our EEG, it is impractical to localize the origins of these activities directly using the EEG data. Therefore, instead of searching for the source locations (i.e., solving the inverse problem), we inform the DCM analysis of EEG by using previous fMRI results of source locations and the connection architecture (for a review, see Kiebel et al., 2009). This is based on the fact that there is a congruity between fMRI activity and the corresponding magnetoelectrical dipoles when measured spontaneously (Korvenoja et al., 2006; David et al., 2008; Lenartowicz et al., 2016). By employing the fMRI priors, the analytic strategy (fMRI-informed DCM analysis) provides a unique solution for the data features (for a detailed discussion on the question of model specification, see our previous work, Chen et al., 2008). Through iteratively comparing the acquired EEG data to the synthetic scalp data generated from the pre-assumed sources and the connection parameters (i.e., the plausible model) *via* the lead field matrix, the source locations and the connection parameters can be optimized on a single subject basis such that the difference between the synthetic data and the EEG is minimal. It should be noted that the low spatial resolution EEG are inadequate to detect the activities from deep or local small sources, but receive the most vigorous features from the cortical areas. Hence, we limited our analysis to only cortical sources. In addition, a few patterns are not shared by fMRI and EEG (David et al., 2008; Lenartowicz et al., 2016) and cannot be used in the analysis. Importantly, the analytic results are always conditional on the hypothesis being tested (i.e., the plausible models) and the experimental settings. An appropriate hypothesis constitutes a trustworthy result. Studies that employ different tasks, devices and plausible models may lead to different results.

In summary, visual WM training altered the feedforward and feedback connections under the ECN and DAN, whereas the practice engaged the parietal-frontal network for P300 generation. Importantly, the results of N160 amplitude increase and the right MFG to SFG suppression after training suggest that only training can modulate the inhibitory machinery for cognitive control.

### Limitations

First, because we recruited only 20 participants and observed a substantial between-group difference in the winning model, it is possible that the results reveal only the features of small samples. Future studies with an adequate number of participants may assist in clarifying the training-specific neuroplasticity. Second, it was suggested that the task design is important in the interpretation of the results (Morrison and Chein, 2011). In this study, the used task may be too easy to fully probe the training effects, because all participants performed well after three repetitions at W2 (accuracy >90) and the mean accuracy did not improve from W2 to W3 in both groups, indicating the ceiling effects of simple tasks. As a result, only minor behavioral differences between the two groups can be established. A more complex task, such as dual visual n-back tasks (Schneiders et al., 2011; Kundu et al., 2013; Thompson et al., 2016) may help reveal the behavioral differences and the neuronal correlates after WM training and practice. Finally, as we did not give any training to the control group, the fact that the WM group have done something more may contribute to the observed between-group differences to some degree in this study. Further study with an active control group can help to solve this confounding.

In conclusion, the connection alternation induced by WM training differs from that induced by WM practice. The WM training effects were mediated by feedforward and feedback connections under the ECN and DAN, whereas the WM practice effects were most manifested in the parietal-frontal network for P300 generation. Our findings provide a reference for distinguishing the visual WM training and practice effects by the underlying neuroplasticity.

## Ethics Statement

The study was approved by the Research Ethics Committee of National Taiwan University (approval number: 201311ES020) in accordance with the ethical principles of the Declaration of Helsinki and all subjects have signed an informed consent form.

## Author Contributions

C-CC and W-JW: conception and design of the study. J-CK and C-CC: acquisition and analysis of data. C-CC, J-CK and W-JW: drafting the manuscript and figures.

## Conflict of Interest Statement

The authors declare that the research was conducted in the absence of any commercial or financial relationships that could be construed as a potential conflict of interest.

## Footnotes

^1^http://www.gyrigym.com/tw/news/index.php
